# Supplementary material for: Ambipolar to Unipolar Conversion in C70/Ferrocene Nanosheet Field-Effect Transistors
Source: Nanomaterials (Basel). 2023 Sep 1;13(17):2469. doi: 10.3390/nano13172469 (PMC10490395; doi:10.3390/nano13172469)
Supplement: Supplementary file 1 [file nanomaterials-13-02469-s001.zip › nanomaterials-2532676-supplementary.pdf]

nanomaterials

Article

Ambipolar to Unipolar Conversion in C_{70}/Ferrocene Nanosheet Field-Effect Transistors

Dorra Mahdaoui [1,2,*], Chika Hirata [1], Kahori Nagaoka [1], Kun'ichi Miyazawa [3], Kazuko Fujii [1], Toshihiro Ando [1], Manef Abderrabba [2], Osamu Ito [1], Shinjiro Yagyu [4], Yubin Liu [5], Yoshiyuki Nakajima [5], Kazuhito Tsukagoshi [6] and Takatsugu Wakahara [1,*]

1 Electronic Functional Macromolecules Group, Research Center for Macromolecules and Biomaterials, National Institute for Materials Science, 1-1 Namiki, Tsukuba 305-0044, Ibaraki, Japan; chikapanman3@gmail.com (C.H.); asagi12@gmail.com (K.N.); fujii.kazuko@nims.go.jp (K.F.); ando.toshihiro@nims.go.jp (T.A.); osamu.ito.b7@tohoku.ac.jp (O.I.)

2 Laboratory of Materials, Molecules and Applications, Preparatory Institute for Scientific and Technical Studies, University of Carthage, B.P. 51, La Marsa 2075, Tunisia; mohamedabdelmanef.benabderrabba@ipest.ucar.tn

3 Department of Industrial Chemistry, Faculty of Engineering, Tokyo University of Science, 6-3-1 Niijuku, Katsushika-ku, Tokyo 125-8585, Japan; miyakuni@rs.tus.ac.jp

4 Nano Electronics Device Materials Group, Research Center for Electronic and Optical Materials, 1-1 Namiki, Tsukuba 305-0044, Ibaraki, Japan; yagyu.shinjiro@nims.go.jp

5 RIKEN KEIKI Co., Ltd., 2-7-6, Azusawa, Itabashi-ku, Tokyo 174-8744, Japan; yb-ryu@rikenkeiki.co.jp (Y.L.); y-nakajima@rikenkeiki.co.jp (Y.N.)

6 International Center for Materials Nanoarchitectonics (WPI-MANA), National Institute for Materials Science, 1-1 Namiki, Tsukuba 305-0044, Ibaraki, Japan; tsukagoshi.kazuhito@nims.go.jp

* Correspondence: dorra.mahdaoui@ipest.ucar.tn (D.M.); wakahara.takatsugu@nims.go.jp (T.W.)

Supporting Infromation

1. Experimental Details

The C_{70}/Fc nanosheets were synthesized following the method described in the literature [1]. Firstly, a toluene solution was prepared and saturated with C_{70}. Afterward, the solution underwent filtration to eliminate any remaining undissolved C_{70} powder. Following that, ferrocene powder was introduced into the C_{70}-saturated toluene solution, which was subsequently ultrasonicated in an ice water bath. In a gradual manner, IPA was gently added to the toluene solution, and the resulting mixture was manually shaken. The resulting mixture was then stored at room temperature for a duration of 24 hours, facilitating the growth of C_{70} /Fc nanosheets. The nanosheets obtained in this study were thoroughly characterized. Visible/near infrared diffuse reflectance spectroscopy was utilized to investigate the optical properties of the nanosheets. The Photoacoustic Yield Spectrum Analysis (PYSA) was performed using the advanced RIKEN KEIKI AC-3 system, equipped with a monochromated D2 lamp. These comprehensive characterization techniques provide valuable information about the nanosheets, contributing to a better understanding of their potential applications.

2. Fabrication of FET devices

The fabrication process of the FET (Field-Effect Transistor) devices involved depositing solutions of C_{70}/Fc nanosheets onto prepatterned substrates. The prepatterned substrates consisted of heavily doped Si wafers as gate electrodes, with a gate dielectric layer of thermally oxidized SiO2 measuring 300 nm in thickness. Gold electrodes were used as the source and drain, with channel lengths ranging from 2-10 μm and widths of 10,000 μm. The preparation of these substrates was conducted at the NIMS NAMIKI Foundry. Electrical transport properties of the devices were measured using a semiconductor analyzer (Agilent B2902A, Agilent E5272A) inside a controlled environment glove box.

References

1. Osonoe, K.; Kano, R.; Miyazawa, K.; Tachibana, M. Synthesis of C70 two-dimensional nanosheets by liquid–liquid interfacial precipitation method. *J. Cryst. Growth.* **2014**, *401*, 458–461.

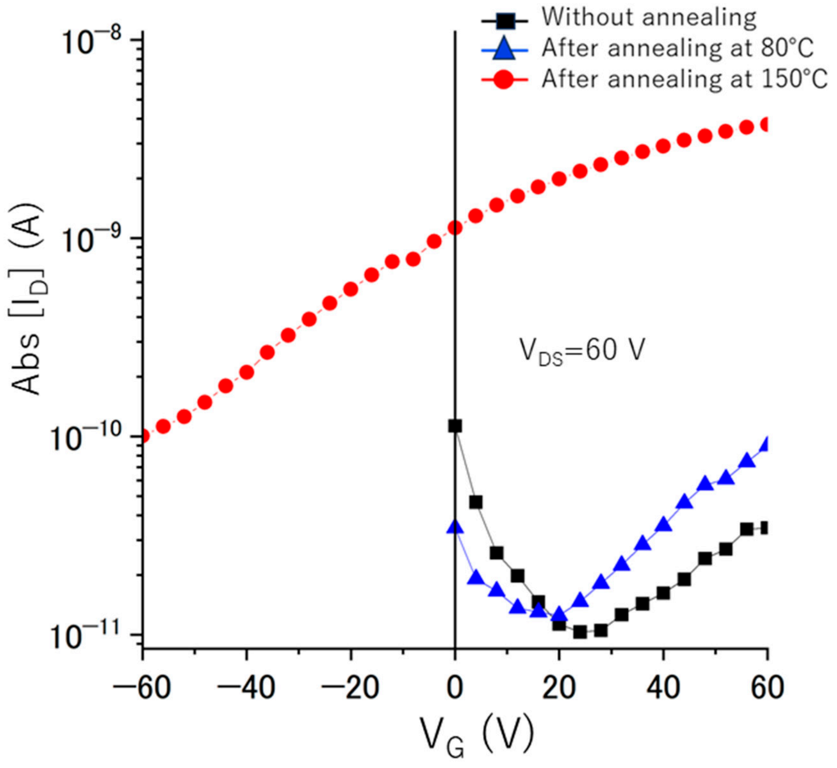

Fig. S1. Transfer (I_D-V_G) characteristics of C_{70}/Fc nanosheets in the dark after annealing.
